# Influence of Source Parameters on the Polarization Properties of Beams for Practical Free-Space Quantum Key Distribution

**DOI:** 10.3390/e23091224

**Published:** 2021-09-17

**Authors:** Tianyi Wu, Qing Pan, Chushan Lin, Lei Shi, Shanghong Zhao, Yijun Zhang, Xingyu Wang, Chen Dong

**Affiliations:** 1 Information and Communication College, National University of Defense Technology, Xi’an 710006, China; wutianyi13@nudt.edu.cn (T.W.); panqingddl@163.com (Q.P.); lcs8409@sina.com (C.L.); gfkd_zyj@163.com (Y.Z.); 2 School of Information and Navigation, Air Force Engineering University, Xi’an 710077, China; slfly2012@163.com (L.S.); zhaoshangh@aliyun.com (S.Z.); 3 Graduate Institute, Rocket Force University of Engineering, Xi’an 710025, China

**Keywords:** QKD, polarization properties, free space, channel modeling

## Abstract

Polarization encoding has been extensively used in quantum key distribution (QKD) implementations along free-space links. However, the calculation model to characterize channel transmittance and quantum bit error rate (QBER) for free-space QKD has not been systematically studied. As a result, it is often assumed that misalignment error is equal to a fixed value, which is not theoretically rigorous. In this paper, we investigate the depolarization and rotation of the signal beams resulting from spatially-dependent polarization effects of the use of curved optics in an off-axis configuration, where decoherence can be characterized by the Huygens–Fresnel principle and the cross-spectral density matrix (CSDM). The transmittance and misalignment error in a practical free-space QKD can thus be estimated using the method. Furthermore, the numerical simulations clearly show that the polarization effect caused by turbulence can be effectively mitigated when maintaining good beam coherence properties.

## 1. Introduction

Quantum key distribution (QKD) can provide an information theoretic security to share keys between two distant parties [1,2,3]. Since its initial proposal, the QKD is believed to be one of the technologies in quantum information science to reach the applications of the quantum network. The rapid development of the ground-based QKD scheme and implementation has resulted in the communication range reaching as far as 511 km with modern technology in the optical fiber channel [4]. Scaling quantum communication protocols over long distances is very challenging due to the losses experienced during the propagation inside the optical fibers. Attempting to overcome the limits imposed by losses, there has been increasing interest [5,6,7,8,9] in implementing QKD through free-space channels.

At present, the polarization-encoded photons from a weak coherent pulse (WCP) source have been experimentally demonstrated to be more suitable than the phase-encoding scheme in free-space QKD [10]. However, in some previous theoretical studies [11,12,13], the fluctuation properties of quantum polarization through a turbulent atmosphere have been ignored when estimating the performance of polarization-encoding QKD, where the misalignment error is only assumed as a constant result (i.e., 1.5%) from the experiment [9], which is not theoretically rigorous.

However, in a free-space polarization-encoded QKD, the spatially-dependent polarization effects resulting from the use of curved optics in an off-axis configuration will lead to decoherence of the quits and rotation of the polarization angle [14,15,16], which will directly increase the misalignment error and further influence the performance of the key rate in the polarization-encoded QKD. Specifically, when estimating the key rate of the QKD system, the gain and the error rate of single photon states are required to be estimated in the decoy-state QKD method [17,18,19], where the standard fiber-based channel model [20] uses some assumed channel parameters. In this paper, we propose a method based on the cross-spectral density matrix (CSDM) [21] to show a more practical free-space QKD key rate estimation. Compared with other methods, our proposal exploits the unified theory of coherence and polarization to model the spatially-dependent polarization effects on the properties of quantum signals in propagation. Moreover, the fluctuation properties of quantum polarization through turbulent atmosphere can also be characterized by our method.

## 2. Propagation Modeling and Key Rate Estimation

### 2.1. Propagation Modeling

**Figure 1:**
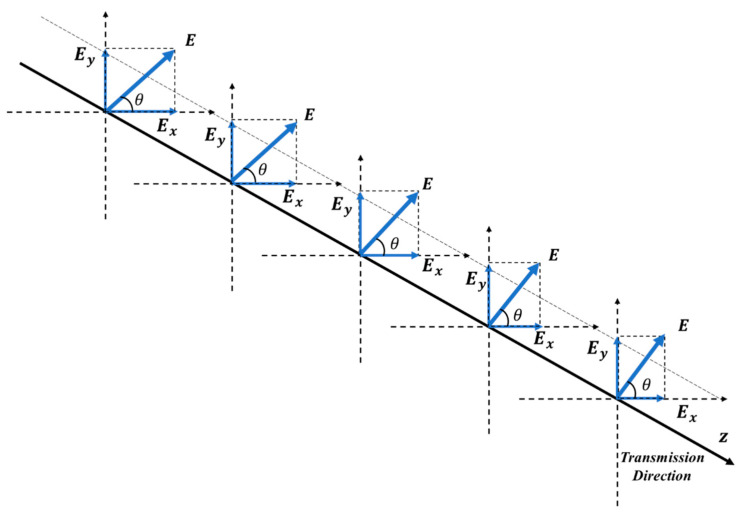
The rotation of polarization angle θ as the beam propagates.

In some practical applications, if one is to implement a QKD over a free-space channel between a satellite platform and a ground station, the turbulence may cause the beam depolarization and the rotation of polarization angle, as Figure 1 shows. This causes continuous phase modulations on the optical beam, and thereby leads to random refraction and diffraction effects, imposing distortions on the optical beam as it propagates through the atmospheric channel.

To model the propagation properties of quantum signals in a free-space channel, we suppose that a coherent Gaussian Schell-model (GSM) is assumed as the quantum signal beam [22], which is generated by the Alice located in the source plane z=0 and propagates along the transmission direction z=0 into the detection Bob (the received plane z>0). In our model, the use of the elements of cross-spectral density matrix (CSDM) in the x and y coordinates $W_{ij}\left(\rho_{1},\rho_{2};0\right),\;i=x,y$ is exploited to show the changes in the polarization of quantum light propagation, which can be expressed as [23]

(1) $$\begin{array}{cc} W_{ij}(r_{1},r_{2};0) & =\langle E_{i}(r_{1};0)E_{j}^{*}(r_{1};0)\rangle \\ & =A_{i}A_{j}\exp[-(\frac{r_{1}^{2}}{4w_{0i}^{2}}+\frac{r_{2}^{2}}{4w_{0j}^{2}})]B_{ij}\exp(-\frac{{\left|r_{1}-r_{2}\right|}^{2}}{2\delta_{ij}^{2}}),i,j=x,y \end{array}$$

Here, $E_{i}$ and $E_{j}$ are, respectively, the components of the random optical field in the  x and y directions, and the asterisk represents the complex conjugate. $\;r_{1}$ and $r_{2}$ denote a pair of points with arbitrary transverse position vectors in the source plane z=0, 〈  〉 denotes the ensemble average. In addition, $A_{i}\left(A_{j}\right)=1$ and $\;w_{0i}\left(w_{0j}\right)$ are the amplitudes and the waist radius of beam in the x and  y directions; The coefficient $\;B_{ij}=1,i=j;\left|B_{ij}\right|\leq 1,i\neq j$ is the spectral degree of coherence. Without any loss of generality, $\;\delta_{ij}$ is the coherence length of the source and, therein, $\delta_{xx}\left(\delta_{yy}\right)$ is the waist radius of the GSM beam in the x/y direction, respectively. Subsequently, when the beam propagates along the transmission direction z from the Alice (source plane z=0) into Bob (received detection plane z>0) in atmospheric turbulence channel, the cross-spectral density matrix at z plane can be expressed by the extended Huygens–Fresnel principle [24]

(2) $$\begin{array}{ll} W_{ij}(\rho_{1},\rho_{2};z) & =A_{i}A_{j}{\left(\frac{k}{2\pi z}\right)}^{2}\iint \exp[-(\frac{r_{1}^{2}}{4w_{0i}^{2}}+\frac{r_{2}^{2}}{4w_{0j}^{2}})]\mu_{ij}(r_{1}-r_{2})\\ & \times \exp[-\frac{ik}{2z}({\left|\rho_{1}-r_{1}\right|}^{2}-{\left|\rho_{2}-r_{2}\right|}^{2})]\\ & \times {\langle \exp[\psi (\rho_{1},r_{1})+\psi^{*}(\rho_{2},r_{2})]\rangle }_{\mathrm{at}}dr_{1}dr_{2} \end{array}$$

where k is the optical wave number related to the wavelength λ by k=2π/λ. $\;\psi \left(\rho_{1},r_{1}\right)$ describes the effects of the atmospheric turbulence on the propagation of a spherical wave due to the atmospheric turbulence from the point (r,0) to  (ρ,z). Considering the Kolmogorov spectrum and a quadratic approximation of the 5/3 power law for Rytov’s phase structure function, the ensemble average term in Equation (2) can be rewritten as

(3) $${\langle \exp[\psi (\rho_{1},r_{1})+\psi^{*}(\rho_{2},r_{2})]\rangle }_{\mathrm{at}}=\exp\{-{\left[{\left(\rho_{1}-\rho_{2}\right)}^{2}\right]}^{5/6}/r_{0}^{5/3}\}$$

where $r_{0}$ is the spatial coherence radius of a spherical wave propagation in turbulent atmosphere channel, which can be calculated by

(4) $$r_{0}={\left(0.545k^{2}z\int_{0}^{1}C_{n}^{2}\left(z\cos\alpha \right)dz\right)}^{-3/5}$$

where  α is the zenith angle of the channel and the altitude-dependent generalized refractive-index structure parameter $C_{n}^{2}\left(zcos\alpha \right)$ can be characterized by the ITU-R model [25]. It is also indicated that the phase distortion caused by atmospheric turbulence eventually leads to a beam decoherence [26].

#### 2.1.1. Rotation of Polarization Angle

Based on the unified theory of coherence and polarization, we assume that one of the properties of the quantum states, the polarization angle θ,−π/2≤θ≤π/2, can be represented as the major axis of the polarization ellipse made in the x direction. Thus, the polarization angle θ can be expressed as [27]

(5) θ(ρ;z)=12arctan{2Re[Wxy(ρ,ρ;z)]Wxx(ρ,ρ;z)−Wyy(ρ,ρ;z)}

Thus, without any loss of generality, we consider the characteristic of the received optical signal at the center point $\rho_{0}=\left(0,0,z\right)$, that is, the polarization angle of quantum state at the received detection can be represented as $\theta \left(\rho_{0};z\right)$. Thereby, the rotation of the polarization angle Δθ can be expressed as

(6) $$\;\Delta \theta =\theta (r_{0};0)-\theta (\rho_{0};z)\;$$

#### 2.1.2. Beam Depolarization

The value of cross-spectral density function has the same variation when a beam has the same coherence length in x/y directions. Therefore, we can also conclude that the rotations of the polarization state will not be seriously impacted in a turbulent channel since the values of the wave front phase distortion characterized by the cross-spectral density function caused by atmosphere turbulence, $\psi \left(\rho_{1},r_{1}\right)$, are the same. Nonetheless, the linearly polarized quantum light after transmission will be depolarized in the atmosphere turbulent channel [28]. However, in previous works [29,30], the geometric model has been proposed to calculate the free-space channel loss, η, which is regarded as the channel transmittance, without considering the depolarization effects. Since the distribution of polarized-photon number is still Poissonian [19], we modify the transmittance as $\eta^{\prime }=\eta_{p}\eta$. Here, the ratio $\eta_{p}$ of the effective polarized intensity component to the total intensity of the light beam is assumed as the degree of polarization (DoP), which can be succinctly described by [31]

(7) $$P(\rho ,\rho ;z)=\frac{\sqrt{s_{1}{\left(\rho ,\rho ;z\right)}^{2}{+s}_{2}{\left(\rho ,\rho ;z\right)}^{2}{+s}_{3}{\left(\rho ,\rho ;z\right)}^{2}}}{s_{0}(\rho ,\rho ;z)},\;0\leq P(\rho ,\rho ;z)\leq 1$$

where $s_{0},s_{1},s_{2}$ and $s_{3}$ are the Stokes parameters. Specifically, $s_{0}$ represents the total light intensity, $s_{1},s_{2}$ represents the linearly polarized light component in the  x direction and in the 45-degree direction, respectively.$\;s_{3}$ represents circularly polarized light in the right hand. Furthermore, based on the formula between DoP and CSDM, the DoP can be rewritten as

(8) $$P(\rho ,\rho ;z)=\sqrt{\frac{{\left[W_{xx}(\rho ,\rho ;z)-W_{yy}(\rho ,\rho ;z)\right]}^{2}+4W_{xx}(\rho ,\rho ;z)W_{yy}(\rho ,\rho ;z)}{W_{xx}(\rho ,\rho ;z)-W_{\mathrm{yy}}(\rho ,\rho ;z)}}$$

As in the previous assumption, the DoP of the quantum light beam at the received detection can also be represented as $P\left(\rho_{0};z\right)$.

### 2.2. Key Rate Estimation

In this paper, we consider the decoy-state QKD in the asymptotic case and finite data size, the secure key rate of decoy-state BB84 QKD in the asymptotic case is given by

(9) $$R_{\mathrm{GLLP}}=q\left\{-f_{e}\left(E_{\mu }\right)Q_{\mu }h_{2}\left(E_{\mu }\right)+Q_{1}\left[1-h_{2}\left(e_{1}\right)\right]\right\}$$

where q depends on the implementation (1/2 for the BB84 protocol due to the fact that half of the time Alice and Bob disagree with the bases; if one uses the efficient BB84 protocol q≈1 and $f_{e}$ is the error correction inefficiency function, μ is the intensity of the signal state and $h_{2}$ is the binary entropy function. $Q_{\mu }$ and $E_{\mu }$ are the measured gain and the quantum bit error rate (QBER), while $Q_{1}$ and $e_{1}$ are, respectively, the gain and error rate of single photon states estimated using the decoy-state method.

Here, we follow the standard channel model using a known channel transmittance η for key rate estimation. Specifically, when the photon number i of each pulse follows a Poisson distribution with a WCP source at intensity μ set by Alice, the transmittance, yield $Y_{i}$, gain $Q_{i}$ and QBER $e_{i}$ are

(10) $$\begin{array}{l} \eta_{i}=1-{\left(1-\eta \right)}^{i},\\ Y_{i}\approx Y_{0}+\eta_{i}=Y_{0}+1-{\left(1-\eta \right)}^{i},\\ Q_{i}=Y_{i}\frac{\mu^{i}}{i!}e^{-\mu },\\ e_{i}=\frac{e_{0}Y_{0}+e_{d}\eta_{i}}{Y_{i}}, \end{array}$$

where $Y_{0}$ and $e_{0}$ are the dark count rate and the error rate of background, respectively, and $e_{d}$ is the misalignment error. Then, the overall gain $Q_{\mu }$ and QBER $E_{\mu }$ for the intensity μ are

(11) $$\begin{array}{l} Q_{\mu }=\sum_{i=0}^{\infty }Y_{i}\frac{\mu^{i}}{i!}e^{-\mu },\\ E_{\mu }=\sum_{i=0}^{\infty }e_{i}Y_{i}\frac{\mu^{i}}{i!}e^{-\mu } \end{array}$$

The misalignment error comes from the long-distance QKD experiment reported in Reference [10], and the channel transmittance is assumed as a given value. However, the modified misalignment error $e_{d}=\overset{3}{\underset{k=1}{\sum }}sin^{2}\Delta \theta_{k}$ (*k* = 1, 2, 3) can be acquired when the misalignment error occurs in three channels for the measurement-device-independent (MDI)-QKD protocol in References [32,33]. Thus, we can calculate $e_{d}$ through $e_{d}=sin^{2}\Delta \theta$ in only one channel for our decoy-state BB84 QKD protocol once the rotation of polarization angle Δθ is obtained. Moreover, it can be seen from Equation (7) that, for the linearly polarized quantum light after transmission in a turbulent atmosphere channel, the polarized light component in the total light intensity will be reduced (i.e., $P\left(\rho_{0};z\right)<1$). Thus, the practical transmittance can be expressed as $P\left(\rho_{0};z\right)\eta$.

## 3. Results

**Figure 2:**
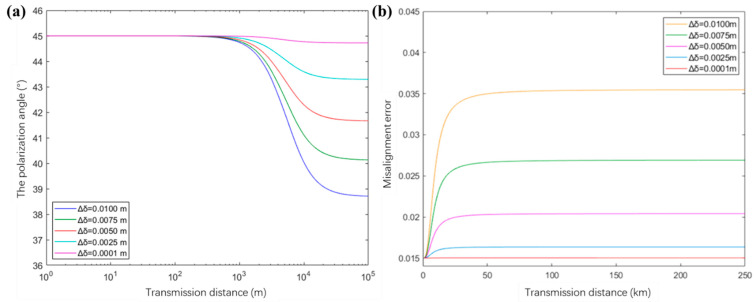
The evolution of the polarization angle (**a**) and misalignment error (**b**) of the 45-degree polarization state versus transmission distance in a free-space channel. Since the misalignment error only depends on the rotation of polarization angle Δθ, the result is same with respect to the −45-degree, and the vertical polarized and horizontal polarized states in the theoretical simulations.

In this section, we put the above models into a simulation program for decoy-state BB84 QKD in a turbulent channel where the refraction structure constant $C_{n}^{2}$ is fixed at 10^−13^. Here, we first estimate the spatially-dependent polarization effects caused by the use of curved optics in an off-axis configuration. The 45-degree polarized state under a coherent beam with the different coherence length (i.e., the difference Δδ between the coherence length $\delta_{xx}$ in the x direction and $\delta_{yy}$ in the y direction) is taken as an example, and the results of the misalignment errors of the beam in the vacuum channel are shown in Figure 2b. It can be observed that one factor that causes the rotation of the polarization angle is the coherence characteristic of the laser beam itself. Once the difference Δδ is close to zero (i.e., the ideal coherent beam), the rotation of the polarization angle is negligible. This is because, if the beam has the same coherence length in x/y directions, the variation in cross-spectral density function is equal according to Equation (2), thus, there are almost no rotations. In this case, the expected misalignment error is consistent with the reported value of 1.5%, which means the polarization misalignment is due to the measurement setting.

**Figure 3:**
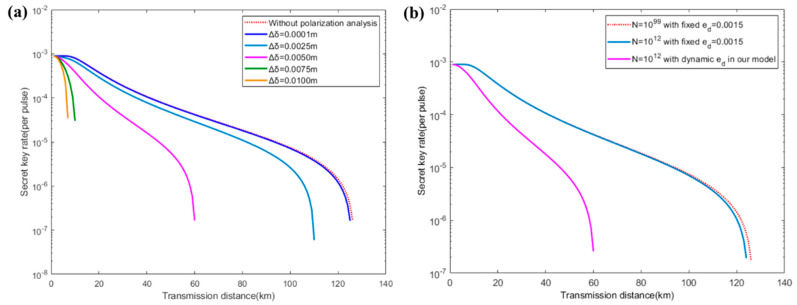
(**a**) Key rate comparison against coherent length difference Δδ in the asymptotic case and (**b**) key rate comparison against finite/infinite data size or fixed/dynamic probability of misalignment error $e_{d}$ in our model.

To perform the influence of polarization analysis on the secret key generation rate under different beam qualities, we plot the comparison results both in the asymptotic case and finite data size. Here, we only fix the signal and decoy intensities to μ=0.3 and ν=0.05. The probabilities of sending them are equal and the probability of sending X basis is 0.5. Other experimental parameters are listed in Table 1. As shown in Figure 3a, the key rate under a coherent beam with Δδ=0.0001 m is very close to the ideal case. As the coherent length difference Δδ of the laser beam increases from 0.0001 m to 0.005 m, the maximum tolerable transmission distance is reduced from over 120 km to approximately 60 km. Moreover, the valid secret key rate is hard to realize over 20 km when a coherent beam with Δδ=0.0075 m or Δδ=0.01 m is applied.

The practical key rates in finite data size are also shown in Figure 3b. We compared the key rate of QKD in the dynamic misalignment errors calculated by our model (blue line) and the fixed misalignment errors (1.5%) reported in the previous works (pink line), respectively, and we took a moderate value Δδ=0.005 m in the range of Δδ=0.0001~0.01 m, as shown in Figure 3a. It can be seen that the maximum tolerable transmission distances are both around ~125 km under infinite data size (*N* = 10^99^) or finite data size (*N* = 10^12^). Compared to the finite length effect, the maximum tolerable transmission distance is reduced to 60 km with a dynamic probability of misalignment error $e_{d}$ in our model, which shows that the probability of misalignment error $e_{d}$ has a severe impact on the key rate. These results highlight the importance of improving the beam quality in the practical implementation of free-space QKD.

## 4. Conclusions

We propose a method where the characteristics of beams in a free-space channel can be calculated by the extended Huygens–Fresnel principle and the coherent Gaussian Schell-model (GSM). Based on the unified theory of coherence and polarization, a general model is proposed for determining the rotation and depolarization effects, which gives more practical results than those in the previous free-space QKD work. We also compare the key rates in both the asymptotic case and the case with finite-size effect, which show a more critical impact on the key rate from the misalignment error than that of the finite-size effect. Furthermore, our numerical simulations show that the polarization effect caused by turbulence can be effectively mitigated when maintaining good beam coherence properties. Hence, it is suggested that the use of a laser beam in free-space QKD experiments should be considered more cautiously. We remark that our method can also be applied to different QKD protocols, including decoy-state BB84 QKD and MDI-QKD. Of course, more parameters of laser sources will be taken into consideration when our method is applied in MDI-QKD, as discussed in References [34,35,36].

## Figures and Tables

**Table 1:** Lists of Necessary Parameters.

Symbol	Name	Value
λ	wavelength of the laser beam	1550 nm
$\omega_{0}$	waist of the laser beam	3.5 cm
N	total number of signals	10^14^
$\eta_{d}$	detection efficiency	50%
$e_{0}$	error probability of dark counts	0.5
$e_{d}$	error probability of optical misalignment	0.015
$\;f_{e}$	Error correction efficiency	1.16
$Y_{0}$	background rate	3 × 10^−6^
∈	security bound	10^−7^

## Data Availability

Data are contained within the article.
